# The effects of treatment via telemedicine interventions for patients with depression on depressive symptoms and quality of life: a systematic review and meta-analysis

**DOI:** 10.1080/07853890.2023.2187078

**Published:** 2023-03-15

**Authors:** Yin-Hwa Shih, Jiun-Yi Wang, Po-Han Chou, Kuan-Han Lin

**Affiliations:** a Department of Healthcare Administration, College of Medical and Health Science, Asia University, Taichung, Taiwan; b Department of Medical Research, China Medical University Hospital, China Medical University, Taichung, Taiwan; c Department of Psychiatry, China Medical University Hsinchu Hospital, Hsinchu, Taiwan

**Keywords:** Telemedicine, depressive symptoms, quality of life, work and social functioning, meta-analysis

## Abstract

**Aim:** The aim of this systematic review and meta-analysis was to identify, evaluate, and synthesize the evidence from studies that have investigated the treatment effect *via* telemedicine interventions on depressive symptoms, quality of life, and work and social functioning in patients with depression.

**Methods:** Six electronic databases (MEDLINE [1916–2021], PubMED [1950–2021], PsycINFO [1971–2021], Scopus [2004–2021], Embase [1972–2021], and CINAHL [1937–2021]) were systematically searched in March 2021. Reference lists of identified articles were hand searched. Randomized controlled trials were included if they investigated the treatment effects *via* telemedicine interventions in patients who had a depression diagnosis. Quality assessment was evaluated using the critical appraisal checklists developed by the Joanna Briggs Institute.

**Results:** Seventeen (17) trials (*n* = 2,394) met eligibility criteria and were included in the analysis. Eleven (11) randomized controlled trials shared common outcome measures, allowing meta-analysis. The results provided evidence that treatment *via* telemedicine interventions were beneficial for depressive symptoms (standardized mean difference= −0.44; 95% CI= −0.64 to −0.25; *p* < .001) and quality of life (standardized mean difference= 0.25, 95% CI −0.01 to 0.49, *p* = .04) in patients of depression. There were insufficient data for meta-analysis of work and social functioning.

**Conclusion:** This study showed the positive effects of treatment *via* telemedicine interventions on depressive symptoms and quality of life in patients with depression and supported the idea for clinical practice to establish a well-organized telepsychiatry system.

**KEY MESSAGES**
- Telemedicine is effective at reducing symptoms of depression.
- Telemedicine can improve quality of life in persons with depression.

## Introduction

The term ‘telemedicine’ encompasses the use of technology to deliver clinical care at a distance. It ensures that people with limited access to care receive help when they need it [1]. With the advancement of telecommunication techniques, telemedicine has gradually been adapted by clinicians [2] to care for people who live at a distance from medical facilities, have weak social networks, and/or are unable to leave their homes for a medical appointment.

Depressive disorder is the leading contributor to the global health-related burden [3]. The risk factors include adversity, personality, neuroticism, and substance abuse. Depression is a mental and behavioral disorder that causes people to experience anhedonia. Symptoms are sadness, cognitive difficulties, changes in appetite, and sleep disorders. These symptoms reduce patients’ quality of life [4,5] and work and social functioning [6,7].

During the outbreak of the COVID-19 pandemic, health services accelerated the global use of telemedicine. Numerous studies have revealed the advantages of telemedicine, such as reducing healthcare expenditure, improving the efficiency of healthcare delivery, and producing good clinical outcomes [8]. Patients with depression require regular mental consultation and deprescribing. Telemedicine is widely adapted in depression care [9] and for acute medical conditions in psychology practice [10–14]. Recent systematic review has reported the effectiveness of video-based psychotherapy in reducing depressive symptoms compared with in-person services [15]. However, an important literature gap still exists in terms of the treatment effects *via* telemedicine interventions quality of life and work and social functioning in patients with depression.

This study aimed to evaluate the treatment effects *via* telemedicine interventions on depressive symptoms, quality of life, and work and social functioning in patients with depression. The present outcomes will provide a reference for psychological practices, to improve the quality of care for patients with depression.

## Methods

This systematic review is reported according to the Preferred Reporting Items for Systematic Reviews and Meta-Analyses (PRISMA) 2020 statement [16]. A protocol for this review was registered on the PROSPERO International prospective register of systematic reviews (Registration No. PROSPERO CRD42021264916).

Six electronic databases (MEDLINE 1916-2021, PUBMED 1950-2021, PsycINFO 1971-2021, SCOPUS 2004-2021, EMBASE 1972-2021, and CINAHL 1937-2021) were searched in March 2021 using combinations of the following terms including American English/British English spelling variations, prefixes/suffixes, and pluralization: depression; depressive disorder; depressive symptoms; telemedicine; telehealth; telepsychiatry; telepsychology; telemental health; digital mental health interventions; web-based information and communication technology; digital psychological intervention; digital communication devices; websites; SMS text messages; emails; smartphone apps; videos; audio files; computer programs; live video; videoconferencing; Symptoms of mental illness; well-being; function; quality of life. Electronic databases were accessed through Asia University, Taiwan. The publication dates or language were not restricted. An example of search strategy is presented as online supplementary material. The additional articles were searched by the reference lists of identified articles.

Two reviewers (JYW, YHS) independently assessed the titles/abstracts of studies for inclusion. Then, full texts of potential studies were screened to determine eligibility by two reviewers (JYW, YHS). Discrepancies between the two reviewers in the study selection processes were discussed for agreement, and a third reviewer (KHL) was consulted if necessary. The criteria for considering studies for this review were the following:

### Types of studies

We included randomized controlled trials (RCTs) (including cluster and cross-over trials). Studies published in English were included. Studies published in non-peer reviewed journals, conference abstracts, letters to the Editor, any forms of literature review, and case studies were excluded.

### Types of participants

Any patients and populations with a depression diagnosis (as diagnosed using any recognized diagnostic criteria) were included.

### Types of interventions

According to the World Health Organization (WHO), telemedicine is defined as ‘the delivery of health care services, where distance is a critical factor, by all health care professionals using information and communication technologies for the exchange of valid information for diagnosis, treatment and prevention of disease and injuries, research and evaluation, and for the continuing education of health care providers, all in the interests of advancing the health of individuals and their communities.’ [17]. We considered all types of telemedicine interventions (including telephone, smartphone, internet-based). Acceptable control interventions were usual care, placebo treatment or wait list controls. We excluded studies in which the control group had, or might have received telemedicine treatments.

### Types of outcome measures

Patient-reported outcome measures of depressive symptoms or quality of life, or work and social functioning were included.

Data extraction and quality assessment were undertaken independently by two reviewers (JYW, YHS) and cross-checked by a third independent reviewer (KHL). The following data items were extracted from included studies: author’s name; year of publication; author and year of publication, country of study, study subject, sample size, intervention group, control group, duration, frequency, outcome measures, and results of outcome measures. Where trial data were not reported or in a form that could not be used in the formal comparisons, we sought further clarification by contacting the authors of included studies. All included trial data were extracted and processed as described in the Cochrane Handbook for Systematic Reviews of Interventions [18]. Methodological quality and bias of each included study was assessed utilizing the critical appraisal checklists developed by the Joanna Briggs Institute (JBI) [19]. JBI critical appraisal checklist for randomized controlled trials have 13 items, and each item was scored as ‘yes’, ‘no’, ‘unclear’, and ‘not applicable.’ Any disagreements were resolved through discussion.

SPSS for Windows statistical software package (SPSS Inc., Version 25, Chicago, IL, USA) was used to analyze the Kappa statistics and agreement, which represented the agreement between reviewers for study selection and quality assessment [20]. Values of the Kappa greater than 0.75 were indicated excellent agreement [18]. Meta-analyses were performed in Review Manager (Review Manager (RevMan) Version 5.3. to identify the treatment effect *via* telemedicine intervention. Only studies which provided pre- and post-intervention data and evaluated outcome of interest in at least two RCTs were included for meta-analysis. Cochran’s Q test and I^2^ were used to assess the heterogeneity among the included studies. The Mantel-Haenszel methods with a fixed-effects model was used in the meta-analyses to pool data from different studies. If heterogeneity was statistically significant (Chi^2^ test, *p* < .05) or I^2^>50%, a more conservative random-effects model was suggested for meta-analysis [18]. The standardized mean differences and 95% confidence intervals (CIs) were computed when the outcome of included studies measured on the different scales. Forest plots were provided to display the mean differences and 95% CIs in the measures between the intervention and control groups. A *p*-value < .05 was considered statistically significant.

## Results

**Figure 1.**
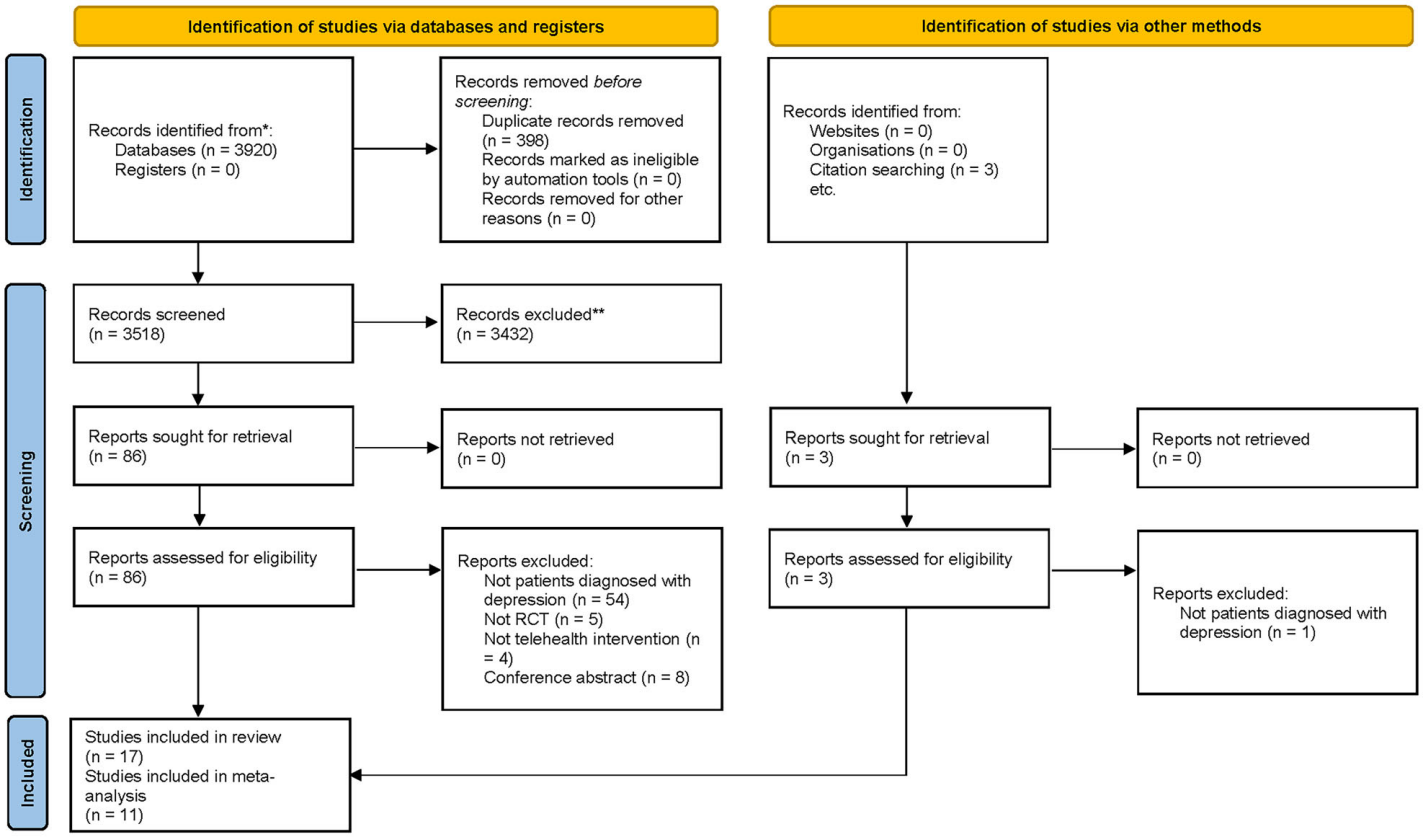
PRISMA flow diagram of study selection process.

Figure 1 presents the PRISMA flow diagram of study selection process. The search of six electronic databases generated 3,920 studies and three additional studies. After removal of duplicate and assessment of eligibility of articles, 17 studies were included for final review, of which 11 studies were entered into meta-analyses. Kappa statistics and agreement between two reviewers on title/abstracts and full text were 0.81 (90% agreement) and 0.73 (84% agreement), respectively. The quality of included studies evaluated by the JBI critical appraisal checklist is shown in online supplementary material.

Seventeen RCTs studies were included, and study characteristics are shown in Table 1. All included studies were published between 2000 and 2020 and written in English. The geographical distribution revealed that of the included studies, eight were conducted in the USA [10,11,22,25,28,31–33], three in Germany [12,13,24], one in Canada [21], one in Korea [23], one in Spain [26], one in Australia [27], one in Chile [29], and one in Sweden [30].

**Table 1.** Participants and intervention programs in reviewed randomized controlled trials.

Author and year	Country	Study subject	Sample size	Comparison: Intervention group	Comparison: Control group	Intervention period/time/type	Outcome measures
Rickhi, 2015 [21]	Canada	Major depressive disorder (DSM IV-TR)	Intervention = Younger13–18yr (*n* = 18) Older 19–24yr(*n* = 16) Control = Younger(*n* = 13) Older(*n* = 16)	Online spirituality informed e-mental health intervention	Waitlist group	8 week	CDRS, HAM-D, SWBS, SIBS, Piers Harris 2, SFSCS
Hunkeler, 2000 [22]	USA	Major depressive disorder (DSM IV-TR)	Intervention= 179 Control = 123 Baseline participants are different from outcome’s (n)	Nurse Telehealth Care	Usual Physician Care	1 to 2 telephone calls per week during 16 weeks Calls were limited to 10 min	HDRS, BDI, SF-12 MCS, SF-12 PCS
Meyer, 2019 [13]	Germany	Diagnosis of active epilepsy with a current depressive disorder (MINI)	Intervention= 100 Control = 100	Internet Cognitive Behavioral Therapy + Care as usual	Care as usual	3‐month, 6‐month, and 9‐month	PHQ‐9, NDDIE, GAD-7, DASS-21, WSAS, QOLIE‐10
Kim, 2018 [23]	Korea	Breast cancer patients with mild to moderate depression (SCID-5-CV)	Intervention = 17 Control = 18	Game group were asked to play the serious game ‘‘Hit the Cancer’’ for at least 30 + typical care	Nongame group received typical care for 3 weeks.	Hit the Cancer’’ game for at least 30 min/day, 5 days/week, for 3 weeks.	BDI, BAI, SRI
Yeung, 2016 [11]	USA	Chinese American Immigrants with MDD (MINI)	Intervention= 97 Control = 93	Telepsychiatry-based culturally sensitive collaborative treatment (T-CSCT)	Treatment as usual (TAU)	6 months	HDRS-17, CGI-S, CGI-I, Q-LES-Q
Beiwinkel, 2017 [24]	Germany	Mild to moderate depression (ICD)	Intervention= 100 Control = 80	Web-based program based on cognitive-behavioral therapy, awareness training, and systemic counseling and therapist support upon request.	Waitlist control	12 week Web-based program	PHQ-9, BDI-II
Place, 2020 [25]	USA	Varying mental health and physical diagnoses	Intervention= 33 Control= 35	Usual care plus the mobile monitoring system	Usual care	6-month	PHQ-9, Schwartz Outcome Scale
Gili, 2020 [26]	Spain	Major depression or dysthymia, older than 18 years, able to understand and read Spanish, with mild or moderate depression (MINI) according to the PHQ-9	Intervention: 1.54 2.54 3.56 control: 57	Healthy lifestyle program + iTAU Mindfulness program + iTAU positive affect promotion program + iTAU	Improved Treatment as Usual (iTAU)	Pretreatment, posttreatment, 6-month, 12- month posttreatment assessments; 4 web-based, individual, and interactive therapeutic modules.	PHQ-9, EQ-VAS, SF-12 MCS, SF-12 PCS, PHI
Newby, 2017 [27]	Australia	Comorbid MDD and DM	Intervention = 42 Control = 49	Internet Cognitive Behavioral Therapy + TAU	TAU	3 months; 6 lessons	PHQ-9, SF-12 MCS, SF-12 PCS
Moreno, 2012 [10]	USA	Self-identified Hispanic patients with a major depressive episode	Intervention = 80 Control = 87	Webcam intervention + TAU	TAU	6 months; 6sessions (20–30 min)	MADRS, PHQ-9, Q-LES-Q, SDS
Dobkin, 2020 [28]	USA	Parkinson disease + depressive disorder diagnosis (DSM-5)	Intervention = 37 Control = 35	Telephone-based cognitive-behavioral treatment (T-CBT) + Enhanced TAU	Enhanced TAU	10 one-hour sessions, weekly for 3 months	HAM-D, BDI, SF-36 MCS
Martinez, 2018 [29]	Chile	Adolescents with MDD	Intervention = 65 Control = 78	Remote collaborative depression care (RCDC) +usual care	Enhanced TAU	3 months	BDI, KIDSCREEN-27
Erikksson, 2017 [30]	Sweden	Diagnosed with depression according to DSM-IV (assessed *via* MINI); aged 18 years and older	Intervention = 52 Control = 38	Internet-delivered cognitive behavioral therapy (ICBT) +TAU	TAU	3 months-12 months	BDI-II, EQ-5D
Anguera, 2016 [31]	USA	Major depressive disorder (SCID; DSM-IV)	Intervention = 12 Control = 10	Enhance cognitive control faculties (EVO) [video game]	Problem solving therapy	4 weeks intervention + 4 weeks clinical management	HAM-D, PHQ-9
Kühn, 2018 [12]	Germany	18–65 years old; major depression or dysthymia according to MINI	Intervention = 34 Control = 34	Video game	Waitlist control group	6 weeks	PHQ-9, BDI
Egedes, 2015, 2016^a^ [32,33]	USA	Veterans aged 60 years or older meeting DSM-IV criteria for major depressive disorder	Intervention = 120 Control = 121	In-home videoconferencing technology	Face-to-face therapy	12 months	GDS, BDI, SF-36

DSM IV-TR: Diagnostic and Statistical Manual of Mental Disorders, fourth edition, text revision; MINI: Mini-International Neuropsychiatric Interview; SCID-5-CV: Structured Clinical Interview for DSM-5 Disorders—Clinician Version; ICD: International Classification of Diseases; MDD: Major Depressive Disorder; DM: Diabetes Mellitus; DSM-IV: Diagnostic and Statistical Manual of Mental Disorders: fourth edition; SCID: Structured Clinical Interview for DSM Disorders; DSM-5: Diagnostic and Statistical Manual of Mental Disorders: Fifth Edition; TAU: Treatment As Usual; iTAU: Improved Treatment as Usual; CDRS-R: Children’s Depression Rating Scale Revised; HAM-D: Hamilton Rating Scale for Depression; SWBS: Spiritual Well-Being Scale; SIBS: Spiritual Involvement and Belief Scale; SFSCS: Six-Factor Self-Concept Scale; HDRS: Hamilton Depression Rating Scale; BDI: Beck Depression Inventory; BDI-II: Beck Depression Inventory-II; SF-12 MCS: Short Form-12 Items Health Survey (Mental Component Summary); SF-12 PCS: Short Form-12 Items Health Survey (Physical Component Summary); PHQ‐9: Patient Health Questionnaire–9; NDDIE: Neurological Disorders Depression Inventory for Epilepsy; DASS‐21: Depression Anxiety Stress Scales–21; GAD-7: General Anxiety Disorder-7; MADRS: Montgomery-Asberg Depression Rating Scale; QOLIE‐10: Quality of Life in Epilepsy–10; Q-LES-Q: Quality of Life Enjoyment and Satisfaction Questionnaire; SF-36 MCS: Short Form-36 Items Health Survey (Mental Component Summary); EQ-5D: EuroQol-5 Dimension; EQ-VAS: EuroQol-Visual Analogue Scale; WSAS: Work and Social Adjustment Scale; BAI: Beck Anxiety Inventory; SRI: Stress Response Inventory; CGI-S: Clinical Global Impressions Scale-Severity of illness; CGI-I: Clinical Global Impressions Scale- Global Improvement; PHI: Pemberton Happiness Index; SDS: Sheehan Disability Scale; GDS: Geriatric Depression Scale.

^a^ Trial on the same patient cohort with different outcome measures reported across distinct peer-reviewed journal articles.

The characteristics of participants in the included studies are shown in Table 1. Ten of the seventeen studies only included participants with diagnosed depression [10,11,21,22,24,29–33]. Four studies recruited participants with comorbid depression and certain diseases, including diabetes mellitus [27], active epilepsy [13], breast cancer [23], or Parkinson’s disease [28]. Two studies included a mixed cohort of participants with depression or dysthymia [12,26]. One study included participants with varying mental health and physical diagnoses [25]. A total of 2,394 participants were included in the RCTs. Among them, 1,286 participants were allocated to the intervention groups, and 1,108 were allocated to the control groups.

All studies included interventions with some forms of telemedicine, and control interventions included usual care [10,11,13,22,23,25,27,30], enhanced usual care [26,28,29], placebo treatment [31], face-to-face behavioral activation [32,33], and waitlist controls [12,21,24]. The frequency and duration of the intervention programs varied between the studies (Table 1). Three studies provided internet cognitive behavioral therapy (ICBT), which was based on a structured web-based CBT approach with strong emphasis on behavioral activation [13,27,30]; three studies provided video/mobile game interventions [12,23,31]; two studies provided internet-based psychological intervention programmes, which included structured interactive sessions [24] and interactive therapeutic modules [26]; two studies used in-home videoconferencing operating *via* the standard telephone service [32,33]; one study provided a Webcam intervention (Internet videoconferencing), which consisted of six monthly Webcam sessions with the psychiatrist [10]; one study provided telephone-based cognitive-behavioral treatment (T-CBT), which comprised telephone-based coaching in how to support and encourage the use of new CBT coping skills between therapy sessions [28]; one study provided remote collaborative depression care (RCDC), which consisted of a 3-month treatment that included continuous remote supervision by psychiatrists through shared electronic health records (SEHR) and phone patient monitoring [29]; one study provided online spirituality informed e-mental health intervention, which was an eight-week online program consisted of eight module to guide participants through an exploration of spiritually informed principles [21]; one study provided nurse telehealth care, which consisted of emotional support and focused behavioral interventions in ten 6-minute calls during 4 months by primary care nurses [22]; one study provided telepsychiatry-based culturally sensitive collaborative treatment (T-CSCT), which included the teleconferencing technology and added a cultural component to the collaborative care model [11]; and one study provided a 6-month usual care plus the mobile monitoring system intervention, which asked participants to downloaded the mobile monitoring application to their smartphones [25]. The length of interventions and follow-up duration ranged from three weeks to 12 months.

The outcome measurements were varied across the seventeen studies. Depressive symptoms were examined in most studies (*n* = 16/17) and measured using Patient Health Questionnaire–9 [PHQ-9] (*n* = 7) [10,12,13,24,26,27,31], Beck Depression Inventory [BDI]/[BDI-II] (*n* = 8) [12,22–24,28–30,32], Hamilton Depression Rating Scale [HAM-D] (*n* = 5) [11,21,22,28,31], Children’s Depression Rating Scale Revised [CDRS-R] (*n* = 1) [21], Neurological Disorders Depression Inventory for Epilepsy [NDDIE] (*n* = 1) [13], Depression Anxiety Stress Scales–21 [DASS-21] (*n* = 1) [13], Geriatric Depression Scale [GDS] [32], and MADRS, Montgomery-Asberg Depression Rating Scale [MADRS] (*n* = 1) [10].

Fifty-eight percent of included studies (*n* = 10/17) measured self-reported quality of life. Five studies assessed health-related quality of life and functioning using the Short-Form Health Survey [SF-12/SF-36] [22,26–28,33]. Two studies also assessed quality of life using the Quality of Life Enjoyment and Satisfaction Questionnaire [Q-LES-Q] [10,11]. One study evaluated health-related quality of life in people with epilepsy using the Quality of Life in Epilepsy–10 items [QOLIE-10] [13]. One study assessed health-related quality of life in adolescents using the KIDSCREEN-27 [29]. Two studies measured quality of life using the EuroQol-5 Dimension [EQ-5D] [30] and EuroQol-Visual Analogue Scale [EQ-VAS] [26], respectively. Only one of the seventeen studies assessed work and social function outcome, and this was undertaken using the Work and Social Adjustment Scale [WSAS] [13], which was designed to measure patients’ perceived functional impairment resulting from a health problem.

**Figure 2.**
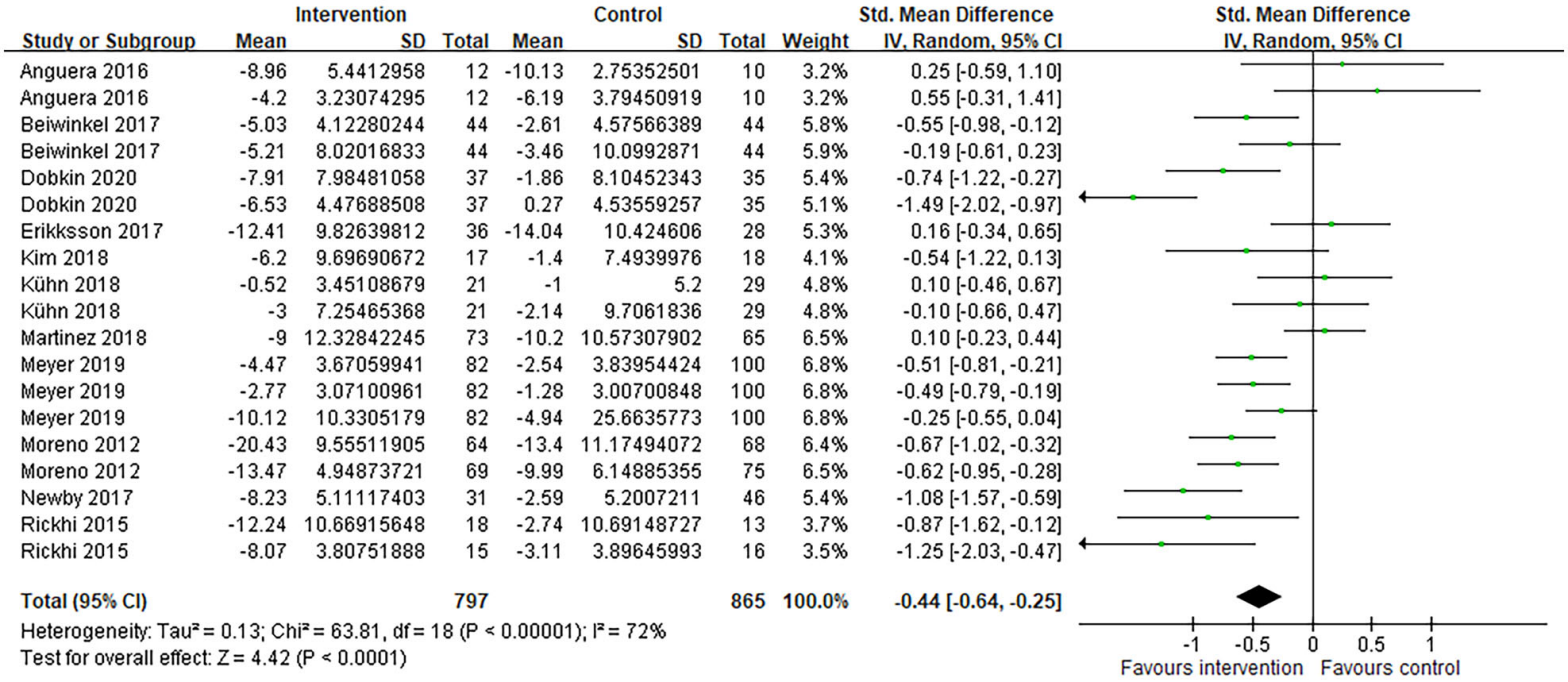
Forest plots of comparison: intervention and control, outcomes: depressive symptoms.

Study results are shown in Table 2. Meta-analysis of the depressive symptoms outcomes after treatment *via* telemedicine intervention showed a significant improvement in depression scores after intervention compared to control. There was a trend towards improved depressive symptoms (standardized mean difference = −0.44; 95% CI= −0.64 to −0.25; *p* < .001). Statistical heterogeneity was present in the comparison between telemedicine and control groups (I^2^ = 72%; *p* < .001) (Figure 2).

**Table 2.** Summary of results on outcome measures in reviewed studies.

Author and year	Measure	*N*	Intervention Group		*N*	Control group	
			Baseline Mean (SD)	Follow-up Mean (SD)		Baseline Mean (SD)	Follow-up Mean (SD)
Depression score							
Rickhi, 2015[21]	CDRS-R Young	18	57.18 (7.93)	44.94 (12.13)	13	61.67 (7.97)	58.93 (12.15)
Rickhi, 2015 [21]	HAM-D Old	15	22.14 (2.52)	14.07 (4.38)	16	20.9 (2.60)	17.79 (4.48)
Meyer, 2019 [13]	PHQ-9	82	14.51 (2.89)	10.04 (4.13)	100	15.27 (3.35)	12.73 (4.19)
Meyer, 2019 [13]	NDDIE	82	16.90 (2.91)	14.13 (3.21)	100	17.30 (2.84)	16.02 (3.15)
Meyer, 2019 [13]	DASS-21	82	28.20 (11.06)	18.08 (9.40)	100	28.84 (9.32)	23.90 (8.52)
Kim, 2018 [23]	BDI	17	25.1 (9.10)	18.9 (10.20)	18	23.00 (8.40)	21.60 (6.00)
Beiwinkel, 2017 [24]	PHQ-9	44	11.53 (4.35)	6.50 (3.85)	44	10.56 (4.53)	7.95 (4.62)
Beiwinkel, 2017 [24]	BDI-II	44	20.07 (7.99)	14.86 (8.05)	44	18.78 (9.84)	15.32 (10.34)
Newby, 2017 [27]	PHQ-9	31	15.95 (5.25)	7.72 (4.96)	46	14.29 (5.25)	11.70 (5.15)
Moreno, 2012 [10]	MADRS	64	29.95 (7.59)	9.52 (10.73)	68	26.86 (8.81)	13.46 (12.57)
Moreno, 2012 [10]	PHQ-9	69	18.41 (4.50)	4.94 (5.30)	75	17.58 (5.12)	7.59 (6.82)
Dobkin, 2020 [28]	HAM-D	37	20.97 (4.40)	14.44 (4.55)	35	21.06 (4.40)	21.33 (4.66)
Dobkin, 2020 [28]	BDI	37	21.00 (7.82)	13.09 (8.14)	35	20.89 (7.81)	19.03 (8.37)
Martinez, 2018 [29]	BDI	73	27.80 (10.20)	18.80 (13.70)	65	27.10 (9.30)	16.90 (11.5)
Erikksson, 2017 [30]	BDI-II	36	25.80 (8.60)	13.39 (10.71)	28	26.50 (9.90)	12.46 (10.88)
Anguera, 2016 [31]	HAM-D,	12	21.50 (3.31)	12.54 (6.28)	10	25.13 (2.62)	15.00 (2.87)
Anguera, 2016 [31]	PHQ-9	12	12.75 (3.52)	8.55 (2.83)	10	15.33 (3.94)	9.14 (3.63)
Kühn, 2018 [12]	PHQ-9	21	8.33 (3.40)	7.81 (3.50)	29	11.45 (5.2)	10.45 (5.2)
Kühn, 2018 [12]	BDI	21	14.76 (7.10)	11.76 (7.40)	29	19.86 (9.9)	17.72 (9.5)
Quality of life							
Meyer, 2019 [13]	QOLIE‐10	98	31.11 (5.68)	33.28 (4.75)	100	30.11 (5.24)	30.91 (5.05)
Newby, 2017 [27]	SF-12 MCS	31	30.22 (10.22)	39.26 (9.80)	46	29.79 (10.01)	32.70 (9.97)
Newby, 2017 [27]	SF-12 PCS	31	40.15 (12.49)	41.10 (11.46)	46	42.94 (11.27)	42.80 (11.12)
Moreno, 2012 [10]	Q-LES-Q	70	0.43 (0.11)	0.72 (0.20)	74	0.44 (0.12)	0.64 (0.19)
Dobkin, 2020 [28]	SF-36 MCS	37	34.06 (11.27)	42.54 (11.38)	35	36.36 (10.99)	38.06 (11.61)
Martinez, 2018 [29]	KIDSCREEN-27	69	3.60 (0.70)	3.50 (0.60)	65	3.60 (0.70)	3.60 (0.8)
Erikksson, 2017 [30]	EQ-5D	36	0.73 (0.14)	0.81 (0.18)	28	0.70 (0.16)	0.81 (0.14)
**Work and social function**							
Meyer, 2019 [13]	WSAS	82	19.54 (7.33)	14.22 (7.75)	100	20.14 (6.58)	18.70 (6.83)

CDRS-R: Children’s Depression Rating Scale Revised; HAMD: Hamilton Rating Scale for Depression; PHQ‐9: Patient Health Questionnaire–9; NDDIE: Neurological Disorders Depression Inventory for Epilepsy; DASS‐21: Depression Anxiety Stress Scales–21; BDI: Beck Depression Inventory; MADRS: Montgomery-Asberg Depression Rating Scale; QOLIE‐10: Quality of Life in Epilepsy–10; SF-12 MCS: Short Form-12 Items Health Survey (Mental Component Summary); SF-12 PCS: Short Form-12 Items Health Survey (Physical Component Summary); SF-36 MCS: Short Form-36 Items Health Survey (Mental Component Summary); Q-LES-Q: Quality of Life Enjoyment and Satisfaction Questionnaire; SF-36 MCS: Short Form-36 Items Health Survey (Mental Component Summary); EQ-5D: EuroQol-5 Dimension; WSAS: Work and Social Adjustment Scale

**Figure 3.**
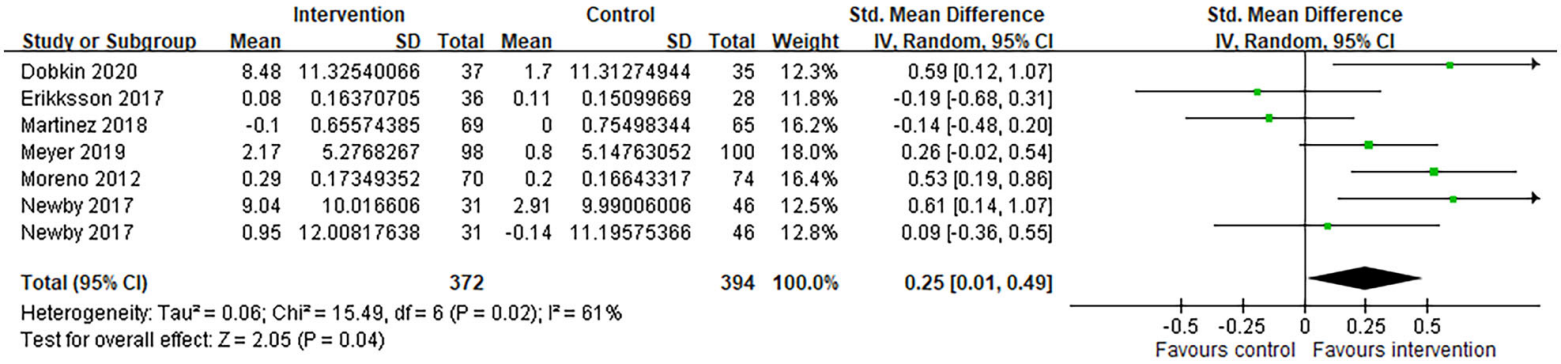
Forest plots of comparison: intervention and control, outcomes: quality of life.

Figure 3 shows a Forest plot for included studies reporting the effect size estimate for treatment *via* telemedicine interventions on quality of life. Pooling results across studies showed a significant trend toward improved quality of life compared to controls. The standardized mean difference was 0.25 (95% CI −0.01 to 0.49, *p* = .04). There was an evidence of statistical heterogeneity (I^2^ = 61%; *p* = .02) (Figure 3).

Insufficient data were provided for meta-analysis of work and social functioning. Only study conducted by Meyer et al. included work and social functioning as one of its outcome measures; thus, this outcome was not included in the meta-analysis [13]. Meyer et al. showed that the intervention group produced clinically significant effects on improvements in social‐occupational impairment, which exceeded improvements observed among control group [13].

## Discussions

This study included seventeen articles from six databases regarding the outcome effects of treatment *via* telemedicine intervention on individuals with depression, and data from eleven articles were extracted for meta-analysis. The forest plot revealed that depressive symptoms improved and quality of life was elevated after the intervention. One of the eligible articles showed evidence that telemedicine improves patients’ social-occupational impairment. Our meta-analysis demonstrated that telemedicine was beneficial in improving depressive symptoms and quality of life in patients with depression. The outcomes of this study support the advantages of telemedicine application in individuals with depression and the concept of clinical practice, establishing a well-organized telepsychiatry system.

The possible reasons for positive outcomes of treatment *via* telemedicine for patients with depression include the following. First, telehealth increases patients’ access to professional help for depression [34]. Common barriers to getting professional help include lack of time, transportation, or the availability of providers in a patient’s local area. Second, treating depression virtually appears to be just as effective as in-person visits [35]. Healthcare providers can easily monitor patients’ clinical conditions by adjusting medication, addressing non-adherence, or providing additional psychotherapy to enhance the quality of patient care [36]. Third, virtual visits were more comfortable. Patients may feel more at ease during an online psychiatrist appointment than during an in-person interview, especially those with severe depression [14].

Telemedicine has advantages and disadvantages for healthcare. Telemedicine benefits patients who live far from healthcare facilities. It also brings benefits to healthcare professionals, who can provide prompt service during patients’ acute conditions and manage patients’ long-term chronic diseases. On the other hand, home-based telemedicine may increase the burden on family caregivers. Family members and patients take more responsibility, and there is greater risk of error in a do-it-yourself homecare scenario. This burden may compel patients and caregivers to seek alternative options for medical services. In addition, telemedicine breaks the boundary between a patient’s public and private life, which affects the significance of the home [37]. Telemedicine lacks the quality of face-to-face human contact. The position of the camera affects the angle of view, unlike face-to-face consultation, which involves facing each other and affects the patient-physician relationship and healthcare quality.

Modern technologies must fill the gaps and limitations in current practice. The future generation of psychiatrists must recognize that the fundamental practice of psychiatry remains based on the patient-physician connection and be aware of the ethical issues and quality of treatment. Physicians must consider an individualized treatment process according to the patient’s characteristics. Therefore, developing a guideline and assessment tool for the quality of care, going beyond the clinical benefit and health outcomes, is needed [38–40].

This study has several limitations. First, the articles excluded patients with suicidal ideation or severe depression. Therefore, the outcome of telemedicine is limited to individuals with mild and moderate depression. Second, we included articles only in English and excluded theses and conference papers, which may have caused language and publication bias. Third, the heterogeneity in the intervention and control groups, regarding program content and duration of the telemedicine components, and statistical heterogeneity in the meta-analyses may limit the interpretation of the results. Fourth, due to the lack of outcome measure data in the RCTs, meta-analysis was limited to eleven studies, which may have affected the strength of the conclusions drawn. This review is further limited by the moderate risk of bias, as most included studies suffer inevitable limitations of blinding patients and assessors to the treatment conditions.

## Conclusion

Treatment *via* telemedicine intervention improved the quality of life and ameliorated the depressive symptoms in patient with mild to moderate depression. Future studies should incorporate with consistent use of telemedicine program and duration of the telemedicine components, given the robust evidence to support the treatment effect *via* telemedicine intervention in this population.

## Supplementary Material

Supplemental Material

Supplemental Material

## Data Availability

Data are available to investigators after request.
