# Supplementary material for: The effects of treatment via telemedicine interventions for patients with depression on depressive symptoms and quality of life: a systematic review and meta-analysis
Source: Ann Med. 2023 Mar 15;55(1):1092–101. doi: 10.1080/07853890.2023.2187078 (PMC10026747; doi:10.1080/07853890.2023.2187078)
Supplement: Supplemental Material [file IANN_A_2187078_SM0540.doc]

Supplementary file 2. Methodological quality of included studies.

| JBI Critical Appraisal Checklist for Randomized Controlled Trials | Newby et al., 2017 | Moreno et al., 2012 | Dobkin et al., 2020 | Martinez et al., 2018 | Eriksson et al., 2017 | Anguera et al., 2016 | Kühn et al., 2018 | Rickhi et al., 2015 | Gili et al., 2020 | Hunkeler et al., 2000 | Meyer et al., 2019 | Kim et al., 2018 | Yeung et al., 2016 | Beiwinkel et al., 2017 | Place et al., 2020 | Egedes et al, 2015 | Egedes et al, 2016 |
| --- | --- | --- | --- | --- | --- | --- | --- | --- | --- | --- | --- | --- | --- | --- | --- | --- | --- |
| 1. Was true randomization used for assignment of participants to treatment groups? | 1 | Ｕ | 1 | 1 | 1 | 1 | 1 | 1 | 1 | 1 | 1 | U | 1 | 1 | 1 | 1 | 1 |
| 2. Was allocation to treatment groups concealed? | 1 | Ｕ | 1 | 1 | 1 | 1 | 1 | 1 | 1 | NA | 1 | NA | 1 | 1 | 1 | 1 | 1 |
| 3. Were treatment groups similar at the baseline? | 1 | 1 | 1 | 1 | 1 | 1 | 1 | 1 | 1 | 1 | 1 | 1 | 1 | 1 | 1 | 1 | 1 |
| 4. Were participants blind to treatment assignment? | NA | NA | NA | NA | NA | 1 | 1 | 1 | 1 | 0 | 0 | NA | NA | 0 | NA | NA | NA |
| 5. Were those delivering treatment blind to treatment assignment? | NA | NA | NA | NA | NA | NA | NA | NA | 1 | 0 | 0 | 0 | 1 | NA | NA | 1 | 1 |
| 6. Were outcomes assessors blind to treatment assignment? | Ｕ | 1 | 1 | 1 | NA | NA | NA | NA | 1 | NA | NA | NA | 1 | NA | NA | 1 | 1 |
| 7. Were treatment groups treated identically other than the intervention of interest? | 1 | 1 | 1 | 1 | 1 | 1 | 1 | 1 | 1 | 0 | 1 | 1 | 1 | 1 | 1 | 1 | 1 |
| 8. Was follow up complete and if not, were differences between groups in terms of their follow up adequately described and analyzed? | 1 | 1 | 1 | 1 | 1 | 1 | 1 | 1 | 1 | 1 | 1 | 1 | 1 | 1 | 1 | 1 | 1 |
| 9. Were participants analyzed in the groups to which they were randomized? | 1 | 1 | 1 | 1 | 1 | 1 | 1 | 1 | 1 | 1 | 1 | 1 | 1 | 1 | 1 | 1 | 1 |
| 10. Were outcomes measured in the same way for treatment groups? | 1 | 1 | 1 | 1 | 1 | 1 | 1 | 1 | 1 | 1 | 1 | 1 | 1 | 1 | 1 | 1 | 1 |
| 11. Were outcomes measured in a reliable way? | 1 | 1 | 1 | 1 | 1 | 1 | 1 | 1 | 1 | 1 | 1 | 1 | 1 | 1 | 1 | 1 | 1 |
| 12. Was appropriate statistical analysis used? | 1 | 1 | 1 | 1 | 1 | 1 | 1 | 1 | 1 | 1 | 1 | 1 | 1 | 1 | 1 | 1 | 1 |
| 13. Was the trial design appropriate, and any deviations from the standard RCT design (individual randomization, parallel groups) accounted for in the conduct and analysis of the trial? | 1 | 1 | 1 | 1 | 1 | 1 | 1 | 1 | 1 | 1 | 1 | 1 | 1 | 1 | 1 | 1 | 1 |

U=Unclear; NA= Not Applicable; 1=Yes; 0=No
