# Supplementary material for: The effects of treatment via telemedicine interventions for patients with depression on depressive symptoms and quality of life: a systematic review and meta-analysis
Source: Ann Med. 2023 Mar 15;55(1):1092–101. doi: 10.1080/07853890.2023.2187078 (PMC10026747; doi:10.1080/07853890.2023.2187078)
Supplement: Supplemental Material [file IANN_A_2187078_SM0533.doc]

### Supplementary Material

### Supplementary file 1. Example of search strategy

### PUBMED was searched using the NIH NLM interface on 25/03/2021 for the period 1950 to March 2021

### The key words and MeSH were both incorporated into the search using combining terms “AND” and “OR”.

1. Population (533,812)

depression OR “depressive disorder” OR “depressive symptoms”

1. Intervention (328,547)

telemedicine OR telehealth OR telepsychiatry OR telepsychology OR telemental health OR “digital mental health interventions” OR “web-based information and communication technology” OR “digital psychological intervention” OR “digital communication devices” OR “smartphone apps” OR websites OR emails OR “SMS text messages” OR videos OR “audio files” OR “computer programmes” OR “live video” OR videoconferencing

1. Outcome (4,939,135)

“Symptoms of mental illness” OR “well-being” OR “function*” OR “quality of life”

### 1 and 2 and 3 (4,054)
